# Bacteriophage classification for assembled contigs using graph convolutional network

**DOI:** 10.1093/bioinformatics/btab293

**Published:** 2021-07-12

**Authors:** Jiayu Shang, Jingzhe Jiang, Yanni Sun

**Affiliations:** Department of Electrical Engineering, City University of Hong Kong, Hong Kong (SAR), China; Key Laboratory of South China Sea Fishery Resources Exploitation and Utilization, Ministry of Agriculture, South China Sea Fisheries Research Institute, Chinese Academy of Fishery Sciences, Guangzhou, Guangdong Province, China; Department of Electrical Engineering, City University of Hong Kong, Hong Kong (SAR), China

## Abstract

**Motivation:**

Bacteriophages (aka phages), which mainly infect bacteria, play key roles in the biology of microbes. As the most abundant biological entities on the planet, the number of discovered phages is only the tip of the iceberg. Recently, many new phages have been revealed using high-throughput sequencing, particularly metagenomic sequencing. Compared to the fast accumulation of phage-like sequences, there is a serious lag in taxonomic classification of phages. High diversity, abundance and limited known phages pose great challenges for taxonomic analysis. In particular, alignment-based tools have difficulty in classifying fast accumulating contigs assembled from metagenomic data.

**Results:**

In this work, we present a novel semi-supervised learning model, named PhaGCN, to conduct taxonomic classification for phage contigs. In this learning model, we construct a knowledge graph by combining the DNA sequence features learned by convolutional neural network and protein sequence similarity gained from gene-sharing network. Then we apply graph convolutional network to utilize both the labeled and unlabeled samples in training to enhance the learning ability. We tested PhaGCN on both simulated and real sequencing data. The results clearly show that our method competes favorably against available phage classification tools.

**Availability and implementation:**

The source code of PhaGCN is available via: https://github.com/KennthShang/PhaGCN.

## 1 Introduction

Bacteriophages (or phages), which mainly infect bacteria, are among the most common and diverse biological entities in the biosphere (McGrath et al., 2007). They regulate the actions of the ecosystem through killing, metabolic reprogramming or gene transfer (Fernández *et al.*, 2018;  Hurwitz and U’Ren, 2016). As a major agent of horizontal gene transfer between bacteria, phages can change the virulence of bacteria and indirectly cause human diseases. There are active studies that use phages for applications such as phage therapy (Loc-Carrillo and Abedon, 2011), disease diagnostics (Bazan et al., 2012; Wang and Yu, 2004) and antimicrobial drug discovery (Liu et al., 2004).

Despite important functions of phages, our understanding of them is still very limited. Metagenomic sequencing, which allows us to obtain total genomic DNA directly from host-associated and environmental samples, has contributed significantly to new phage discovery (Dutilh et al., 2014; Moon et al., 2018, 2020a,b). In particular, metagenomic sequencing allows sequencing of uncultured dark matter of the microbial biosphere, which can contain a large amount of phages (Perez Sepulveda et al., 2016). The advancements of high-throughput sequencing, assembly and contig scaffolding have led to phage-like contigs or genomes from different types of samples. According to the RefSeq database supported by the National Center for Biotechnology information (NCBI), the number of identified phages changed from 1468 in 2015 to 3852 in 2020 in the RefSeq database, which is more than twice of increase. Despite the increase, known phages is just the tip of the iceberg of the virome on the planet (Santiago-Rodriguez and Hollister, 2019). How to automatically and accurately mine phages and assign their taxonomic groups from vast amount of sequencing data remains a challenging problem.

There are two specific challenges for phage classification. First, the phages with known taxa are very limited. The International Committee on Taxonomy of Viruses (ICTV) is responsible for the official virus taxonomy and organizes viruses in order, family, subfamily, genera and species. Current ICTV classification procedures cannot catch up with new phage discovery. For example, one of the phage order named *Caudovirales* has 3691 reference genomes. However, there are more than 1800 new *Caudovirales* sequences found in 2020 that are unclassified into families. Limited labeled genomes pose challenges for both alignment-based and learning-based classification. Second, many phages in different taxa can share protein homologs, which adds ambiguity for alignment-based taxonomic classification. For example, more than 7616 (∼10%) proteins in all annotated phage proteins are shared by phages in different families under *Caudovirales*. In addition, more than 18 970 (∼27%) pairs of highly similar proteins (*E*-value of BLASTP result $<10^{-50}$) are encoded by phages in different families. Therefore, using homology search alone can return ambiguous classification.

In this work, we present a method that automates taxonomic classification for contigs, which are the outputs of assembly. Although taxonomic classification can be conducted on both reads and contigs (Keegan et al., 2016), recombination in viruses can make read-level taxonomic classification difficult. In addition, more distinctive features can be derived from contigs and thus can lead to improved classification accuracy. Current metagenomic assembly tools, such as MEGAHIT (Li et al., 2015), have been extensively tested and can produce quality contigs from complex datasets. Thus, our tool accepts contigs as input. In order to address the aforementioned challenges, we developed a semi-supervised learning framework that incorporated the automatically learned features for each contig via a convolutional neural network (CNN), the protein sequence similarity, and the gene-sharing features between contigs/genomes. Both the unlabeled and labeled sequences were utilized for training in a graph convolutional neural network (GCN). We will demonstrate that the features from the unlabeled sequences (contigs) improve the learning ability and accuracy for phage classification. Below we summarize related work for phage classification.

### 1.1 Related work

Many attempts have been made for phage taxonomic classification. They can be roughly divided into two groups: alignment-based (Aiewsakun *et al.*, 2018; Chibani et al., 2019; Kristensen et al., 2013) and learning-based (Bolduc et al., 2017; Jang et al., 2013; 2019; Rohwer and Edwards, 2002). Alignment-based methods utilize either nucleotide-level or protein-level homology search between query contigs and reference genomes for assigning the taxon for the query. ClassiPhage (Chibani et al., 2019) and Phage Orthologous Groups (POGs) (Kristensen *et al.*, 2013) are two representative alignment-based phage classification tools. POGs extract taxon-specific marker genes and align query sequences against the marker genes using BLASTP. If there are statistical significant alignment for the contigs, the label of the best-aligned marker gene will be assigned to the contigs. ClassiPhage builds a profile Hidden Markov Model (pHMM) for each phage taxonomic group and apply HMM-based alignment for classification. There are two limitations with alignment-based method. First, as genes or proteins can be shared by different taxa, alignment-based method may lead to ambiguous label assignment or return a label with a higher rank using the lowest common ancestor in the phylogenetic tree. Second, as phages are highly abundant and diverse, alignment-based methods are not able to assign taxa for new species that harbor novel proteins or lack quality alignments with the references. For example, under the *Caudovirales* order, 187 006 proteins are named as hypothetical proteins without known family labels. A total of 13 382 proteins from phages released in 2020 do not have BLASTP results with the phages released before 2020. Thus, using only sequence similarity cannot provide ideal resolution.

There are a number of learning-based tools for microbe classification such as the Naïve Bayes classifier (Wang et al., 2007) and CNN (Shang and Sun, 2020). They use either manually derived or automatically learned features to predict taxonomic labels for bacteria or RNA viruses. The most relevant learning-based tool to phage classification is vConTACT 2.0 (Jang *et al.*, 2019), which applies a graph clustering algorithm to assign labels for unknown contigs. In order to leverage gene organization conservation for phage classification, vConTACT utilizes a clustering algorithm to construct a gene-sharing network (Bolduc *et al.*, 2017; Jang *et al.*, 2019). If the reference genomes and contigs are in the same cluster, the labels of the reference genomes will be assigned to those contigs. While these gene-sharing network methods present satisfactory performance on classification of complete genomes, the classification accuracy decreases as the length of the contigs becomes shorter. The decreased performance stems from the fact that short contigs do not contain many proteins and thus do not lead to valid edges in the gene-sharing network. As a result, the clustering algorithms fail to group contigs and reference genomes in the same cluster. Then, no labels will be assigned to these contigs.

Given the enormous diversity of phages and the sheer amount of unlabeled phages, we formulate the phage classification problem as a semi-supervised learning problem. We choose the GCN as our learning model and combine the strength of both the alignment-based and the learning-based methods. First, we utilize DIAMOND-derived sequence similarities between contigs and references (Buchfink *et al.*, 2015) to improve the edge construction process in the gene-sharing network. Second, to handle the situation that short contigs lack gene organization-related features, a CNN-based model is adopted to encode nucleotide information from the sequence. The GCN model allows us to utilize features from both labeled and unlabeled samples in training and thus lead to more accurate and sensitive phage classification. We compared our tool (named PhaGCN) with three state-of-the-art models specifically designed for phage classification: POGs (Kristensen *et al.*, 2013), vConTACT 2.0 (Jang *et al.*, 2019) and ClassiPhage (Chibani et al., 2019). The experimental results demonstrated that PhaGCN outperforms other popular methods.

## 2 Materials and methods

Semi-supervised learning is a machine learning approach that combines a small amount of labeled data with a large amount of unlabeled data during training. The main purpose of using the unlabeled data is to utilize their conjunction information with the labeled data to improve the classification accuracy. Because the number of reference (labeled) phage genomes is small and new (unlabeled) phage contigs are increasing quickly, we formulate the phage classification problem as a semi-supervised learning problem.

One of the semi-supervised learning approaches, named GCN, is based on deep learning. The basic idea of GCN is to apply a convolutional layer on a graph to utilize the features on non-Euclidean structure (Kipf and Welling, 2016). The purpose of the graph convolutional layer is to automatically learn the topological features from the knowledge graph. Then, unlabeled samples/nodes can be represented as the weighted sum of their neighbor samples/nodes features. In biological data analyses, there exist many non-Euclidean structures such as protein topology graph on the supersecondary structure, gene-sharing network and diseases-gene relationship graph. GCN is expected to render high classification performance by employing the structural data. For example, GCN has shown promising results in finding the relationship between long non-coding RNAs and diseases (Alam *et al.*, 2020; Zhao et al., 2020). In phage classification, different phage genomes and contigs can share genes or proteins, which can be encoded in the graph of GCN. In addition, the nodes in GCN can embed automatically learned feature from nucleotide sequences. During the training, convolution is conducted for each node and its neighbors defined by the graph. The learned topological features will then be applied for classifying samples without labels.

**Fig. 1.**
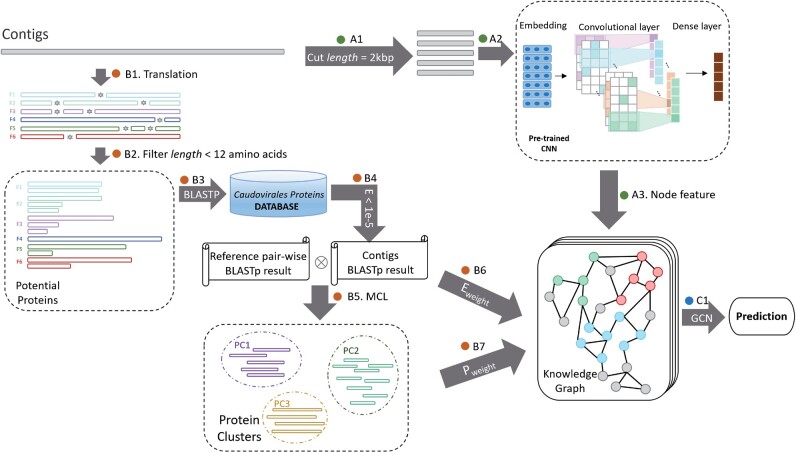
The pipeline of PhaGCN. A1: cut the contigs into 2 kbp segments. A2: feature learning from the inputs using CNN. A3: construct nodes using encoded vectors. B1: contig translation using 6 reading frames. B2: filter short translations (12 amino acids). B3: align contigs against reference database using the DIAMOND BLASTP command. B4: choose the best translated frame for the BLASTP result. B5: use the BLASTP result to construct protein clusters. B6 and B7: define edges based on the sum of the *E_weight_* and *P_weight_*. C1: construct the knowledge graph for GCN

The input to our GCN model is a knowledge graph. There are two key components in the knowledge graph: node encoding and edge construction. The node is a numerical vector learned from contigs using a CNN. The edge encodes features from both the sequence similarity and the organization of genes. Figure 1 contains the major components for node and edge construction. To encode a sequence using a node, a pre-trained CNN is adopted to capture features from the input DNA sequence (A1–A3). The CNN model is trained to convert proximate substrings into vectors of high similarity. The edge construction consists of several steps. We employ a greedy search algorithm to find the best BLASTP results (*E*-value less than 1e-5) between the 6-frame translations of the contigs and the database (B1–B4). Then the Markov clustering algorithm (MCL) is applied to generate protein clusters from the BLASTP result (B5) (Jang *et al.*, 2019). Based on the results of BLASTP (sequence similarity) and MCL (shared proteins), we define the edges between sequences (contigs and reference genomes) using two metrics: *P_weight_* and *E_weight_* (B6–B7). By combining the node’s features and edges (C1), we construct the knowledge graph and feed it to the GCN to classify new phage contigs.

### 2.1 Using CNN to encode input sequences in the knowledge graph

CNN can automatically learn motif-related features for sequence classification (Alipanahi *et al.*, 2015; Seo et al., 2018). Although CNN can be directly applied to phage classification, our experiments will show that using CNN alone cannot render the best classification performance. Thus, we only train the CNN for encoding input contigs.

**Fig. 2.**
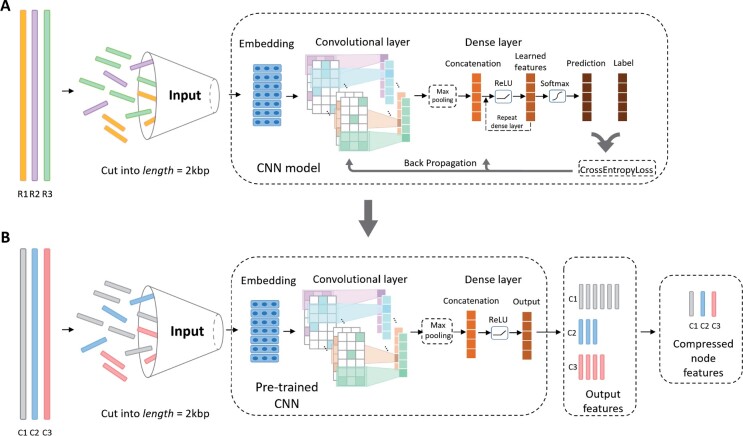
The structures of the CNN model in PhaGCN for training (**A**) and encoding (**B**). R1, R2, R3 are the reference genomes used for training. C1, C2, C3 are the contigs that need to be encoded. In train mode, sequences will be fed to CNN to update parameters during back propagation. In encoding mode. The pre-trained CNN will be used to encode the sequences into numerical vectors. Then these vectors will be adopted as node features in the knowledge graph

As shown in Figure 2, there are two slightly different network structures in the CNN for ‘train mode’ and ‘encoding mode’, respectively. In the train mode (Fig. 2A), we use the reference database to train the CNN model. In the encoding mode (Fig. 2B), the output of the first dense layer in the pre-trained CNN will be used to encode sequences into numerical vectors.

*Train mode*: Because the CNN model can only handle fixed length input, all the inputs will be cut into 2 kbp segments with user-specified stride value (default 50). The segment has the same label as the underlying genome according to the ICTV taxonomic classification.

The CNN model contains three different parts: embedding layer, convolutional layer, and dense layer. The embedding layer is used to convert the DNA sequence into numerical inputs for convolution. There are two major methods for the embedding layer: one-hot embedding and skip-gram embedding (Mikolov et al., 2013). As shown in our previous work of using CNN for classifying RNA viruses (Shang and Sun, 2020), the skip-gram-based embedding can improve CNN’s learning ability. Thus, in this work, we implemented a skip-gram embedding layer that can map proximate k-mers into highly similar vectors. We trained the embedding layer using k-mers and their neighboring (proximate) k-mers so that the embedding layer can learn their adjacent relationship. Specifically, in order to train the embedding layer, we use a 3-mer at position *i* as input and 3-mers located at *i *+* j* as output, where −m≤j≤m. *m* is the hyperparameter that can be specified for the skip-gram model. We employ 100 hidden units in the embedding layer to encode the 3-mers and the output of the embedded vector has 100 dimensions. Thus, each 2 kbp segment is converted into embedded matrix $M\in R^{2,000\times 100}$.

(1) $$Z_{i}(M,w_{\mathrm{conv}})=\mathrm{ReLU}(\sum_{j=1}^{n_{\mathrm{conv}}}w_{\mathrm{conv}}^{j}*M[i:i+d_{1}-1][1:d_{2}]+b)$$

 

(2) $$H^{\left(0\right)}=\mathrm{Maxpool}(Z(M,w_{\mathrm{conv}}))$$

 

(3) $$H^{\left(l+1\right)}=\mathrm{ReLU}(H^{\left(l\right)},w^{\left(l\right)})$$

 

(4) $$\mathrm{output}\;of\;\mathrm{train}\;\mathrm{mode}=\mathrm{SoftMax}(H^{\left(2\right)},w^{\left(2\right)})$$

Then, the embedded matrix *M* will be fed into the convolutional layer. Equation 1 is the convolution function. *b* is the bias term; *d*_1_ and *d*_2_ are the filter sizes. Since the embedded vector has 100 dimensions, $d_{2}=100$. $M[i:i+d_{1}-1][1:d_{2}]$ defines a 2D window size of $d_{1}\times d_{2}$ of the embedded matrix *M*. ReLU is the activation function. The convolutional filters *w_conv_* contain *n_conv_* 2D matrices and $w_{\mathrm{conv}}^{j}$ is the *j*-th filter. We applied filters repeatedly to each possible window of the input embedded matrix to produce a feature map. Then the dense layer is applied to compress the features captured by the convolutional layer as shown in Equations 2 and 3. First, max-pooling (Equation 2) is applied to the feature map to capture the most useful information from the convolutional layer. Second, we use two dense layers with ReLU activation function (Equation 3) to learn and compress the feature map. $H^{\left(l\right)}$ is the feature map in hidden layer *l* and $w^{\left(l\right)}$ is the weight parameters in the *l*-th hidden layer. Since we only has two dense layers, l∈{0,1}. Finally, the SoftMax function (Equation 4) is adopted to generate the prediction. As shown in Figure 2, in the train mode, we employ CrossEntropyLoss to calculate the error between prediction and real label and backpropagate the loss to update the parameters in the model. The detailed parameters are listed in our Github repository.

*Encoding mode*: After training the CNN model, we utilize the pre-trained parameters to convert contigs into numerical vectors. The main difference in the encoding mode is that we only use the output of the first dense layer as the learned feature rather than using the SoftMax function for prediction. Equation 5 shows the equation to convert *x* (an input 2 kbp segment) into the output of the first dense layer. If a contig is cut into multiple segments of length 2 kbp, we will conduct vector addition for all the segments’ outputs and divide it by the number of segments. Thus, contigs of different lengths are always converted into vectors of the same size (determined by the units of the dense layer, default 512).

(5) $$\mathrm{Out}(x)=\mathrm{ReLU}(\mathrm{pool}(Z(M,w_{\mathrm{conv}}),w^{\left(0\right)})$$

### 2.2 Construction of the edge in GCN

The edges connect nodes that are likely in the same taxonomic group. We define the edge by incorporating both the number of shared protein clusters and also the average protein similarity between two sequences. Intuitively, if two sequences share a large number of common protein clusters with high similarity, they tend to belong to the same taxa. In order to quantify the significance of two sequences sharing *c* common proteins, we first define protein clusters. A pair of proteins from two sequences is called a shared protein if they are in the same protein cluster.

#### 2.2.1 Construction of the protein cluster

We follow the idea in Bolduc *et al.* (2017) and Jang *et al.* (2019) to construct protein clusters. We start by extracting proteins from all sequences. For the genomes in the reference database, proteins are downloaded from NCBI RefSeq. For the input contigs, DNA sequences are translated into amino acid sequences using six reading frames. We employ DIAMOND to conduct all-against-all pairwise alignment between contigs’ 6-frame translations and proteins encoded by the genomes. If there are multiple alignments for different reading frames of a contig, only the best frame is kept. Then we create a weighted graph where the node is a protein sequence in the contig or genome and the edge represents an alignment with *E*-value less than a threshold. The edge weight is the *E*-value. Then protein clusters are subsequently identified using the MCL. Finally, clusters that contain at least two proteins will be kept.

#### 2.2.2 Definition of the edges

The edge is defined by computing two metrics: *P_weight_* and *E_weight_*. *P_weight_* is adopted to calculate the expected number of sequences sharing at least an observed number of common proteins (i.e. *c* proteins). Following vConTACT (Bolduc *et al.*, 2017), by assuming that each of the *n* protein clusters has the same chance to be chosen, we compute the probability that any two sequences containing *a* and *b* protein clusters share at least *c* clusters in Equation 6. Equation 7 then computes the expected number of sequence pairs with at least *c* common proteins out of $\left(\begin{array}{c} N\\ 2 \end{array}\right)$ sequence pairs, where *N* is the number of sequences (contigs and reference genomes). With increase of *c*, *P* in Equation 6 becomes small enough to return a positive *P_weight_*.

(6) $$P(y\geq c)=\sum_{i=c}^{\min(a,b)}\frac{\left(\begin{array}{c} a\\ i \end{array}\right)\left(\begin{array}{c} n-a\\ b-i \end{array}\right)}{\left(\begin{array}{c} n\\ b \end{array}\right)}$$

 

(7) $$P_{\mathrm{weight}}=-\log(P(y\geq c)\times \left(\begin{array}{c} N\\ 2 \end{array}\right))$$

While *P_weight_* is used to evaluate whether two sequences share a significant number of common proteins, *E_weight_* is adopted to calculate the sequence similarity using alignments’ *E*-values. For two sequences *A* and *B* with *N_c_* shared proteins, we first define *S*(*A*, *B*) in Equation 8, which is the arithmetic mean of the *E*-values of *N_c_* alignments. *S*(*A*, *B*) has a small value only when all the shared proteins have significant *E*-values, which helps reduce false edge construction for short contigs. For each genome *A*, S(A,A′) is ranked for all A’s adjacent nodes A′. Users can decide how many edges to keep by specifying a threshold. By default, we only keep the top five edges for each genome *A*. For all the kept edges, $E_{\mathrm{weight}}=S(A,B)$.

(8) $$S(A,B)=\{\begin{array}{cc} 0, & if\;no\;\mathrm{alignment}\;\mathrm{result}\\ -\log(\frac{\sum_{}^{}{}_{i=0}^{N_{c}}e_{\mathrm{value}}(i)}{N_{c}}), & \mathrm{otherwise} \end{array}$$

*Edges in the knowledge graph*: The final edge in the knowledge graph is defined based on the sum of *P_weight_* and *E_weight_*. An edge is defined when the sum is above a threshold *τ*, which is 1 by default (Equation 9). It connects two sequences with enough common proteins of high similarity. Usually, as long contigs share more proteins with the reference genome database, *P_weight_* tends to big enough for creating edges between the long contigs and the knowledge graph. However, short contigs have fewer shared proteins and thus we use *E_weight_* to examine whether the shared proteins have significant similarities with references for creating an edge. If a contig has no edge connecting to the knowledge graph, PhaGCN will not output a prediction. Only contigs in the knowledge graph will be fed to the GCN for training and prediction.

(9) $$\mathrm{Edge}=\{\begin{array}{cc} 1, & if\;P_{\mathrm{weight}}+E_{\mathrm{weight}}>\tau \\ 0, & \mathrm{otherwise} \end{array}$$

### 2.3 The GCN model

After constructing the knowledge graph, we train a GCN to assign labels for all unlabeled contigs.

(10) $$H^{\left(l+1\right)}=\mathrm{ReLU}({\tilde{D}}^{-\frac{1}{2}}\tilde{G}{\tilde{D}}^{\frac{1}{2}}H^{\left(l\right)}W^{\left(l\right)})$$

 

(11) $$\mathrm{Out}=\mathrm{SoftMax}(H^{\left(2\right)}W^{\left(2\right)})$$

**Fig. 3.**
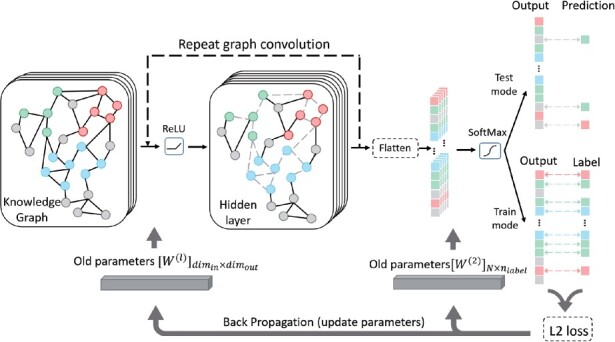
The structure of the GCN model in PhaGCN. Both feature from labeled (green, red and blue color) and unlabled samples (gray color) will be used in training and prediction. In train mode, only labeled samples will be utilize to calculate the loss and update the parameters. In test mode, the SoftMax function will be applied to generate prediction for unlabeled samples

The basic concept of graph convolutional layer is shown in Equation 10. Suppose we have N sequences (nodes) in the knowledge graph. *G* is the $R^{N\times N}$ adjacency matrix of the knowledge graph and *I_N_* is an $R^{N\times N}$ identity matrix. $\tilde{G}$ is calculated with $\tilde{G}=G+I_{N}$. $\tilde{D}$ is the $R^{N\times N}$ diagonal matrix calculated with (Tex translation failed). $H^{\left(l\right)}$ is the feature map in the *l*-th hidden layer; $H^{\left(0\right)}$ is the node feature matrix; and $W^{\left(l\right)}$ is a matrix of weight parameters. After the graph convolutional layer, we apply a dense layer and use the SoftMax function to calculate the output matrix $\mathrm{Out}\in R^{N\times n_{\mathrm{label}}}$ (Equation 11). Because we have two graph convolutional layers and one dense layer in our model, l∈{0,1}. The output dimension *n_label_* is decided by the number of classes in the database. As shown in Figure 3, only the labeled samples will be used to calculate the loss in the training process. We adopt L2 loss to calculate the error between prediction and the labeled samples and back propagate the loss to update the weight parameters. After training the GCN model, we freeze the parameters and use the SoftMax value of the unlabeled samples to assign their labels in the test mode. The detailed parameters are listed in our Github repository.

## 3 Result

### 3.1 Data and performance metrics

We demonstrate the performance of PhaGCN on classifying contigs in families under *Caudovirales*, which is an order containing the majority of known phages from RefSeq (95.8% of total phage reference genomes). We downloaded the *Caudovirales* reference genomes from the NCBI RefSeq database. As shown in Table 1, there are 3639 genomes from eight different families. As the lower ranks contain few genomes in each group, we focus on family-level classification in the experiment.

**Table 1.** Eight families under *Caudovirales*

Name	Number of genomes
*Ackermannviridae*	63
*Autographiviridae*	378
*Demerecviridae*	87
*Drexlerviridae*	112
*Herelleviridae*	136
*Myoviridae*	775
*Podoviridae*	337
*Siphoviridae*	1805

#### 3.1.1 Data and experiment design

PhaGCN was tested on both simulated and real sequencing data. For the simulated data, we applied two different methods to generate contigs with known labels: (1) randomly sample contigs from the reference genome; (2) simulate reads with ART-Illumina (Huang *et al.*, 2012) and run MEGAHIT (Li *et al.*, 2015) to assemble contigs. After validating PhaGCN on simulated data with known ground truth, we downloaded two real sequencing datasets from NCBI SRA and evaluated PhaGCN on assembled contigs. As phages are highly abundant in marine environment samples (Perez Sepulveda *et al.*, 2016), we tested PhaGCN on virus-like contigs from 71 metagenomic data sets that are sequenced from oyster. We recorded macro-accuracy, macro-recall and macro-precision for each experiment (Equations 12–14) when the ground truth can be derived. *N_class_* is the total number of classes. *TP* is the True positive, *TN* is the True negative, and *FN* is the false negative. *Acc_i_* is the accuarcy of class *i*. Except for CNN, each tool can output either a family label or no label at all (no prediction). For each class, if its positive samples have no predictions, they are counted as *FN*. If its negative samples have no predictions, they are counted as *TN*. As macro-average will compute each metric independently for each class and then take the average, these metrics treat all classes equally.

(12) $$Acc_{\mathrm{macro}}=\frac{\sum_{i=0}^{N_{\mathrm{class}}}Acc_{i}}{N_{\mathrm{class}}}$$

 

(13) $$\mathrm{Precisio}n_{\mathrm{macro}}=\frac{\sum_{i=0}^{N_{\mathrm{class}}}P\mathrm{recisio}n_{i}}{N_{\mathrm{class}}}=\frac{\sum_{i=0}^{N_{\mathrm{class}}}\frac{TP_{i}}{TP_{i}+FP_{i}}}{N_{\mathrm{class}}}$$

 

(14) $$\mathrm{Recal}l_{\mathrm{macro}}=\frac{\sum_{i=0}^{N_{\mathrm{class}}}R\mathrm{ecal}l_{i}}{N_{\mathrm{class}}}=\frac{\sum_{i=0}^{N_{\mathrm{class}}}\frac{TP_{i}}{TP_{i}+FN_{i}}}{N_{\mathrm{class}}}$$

The main purpose of PhaGCN is to classify new phages that do not have reference genomes in the training data. When the training and testing data share common genomes, high accuracy may be attributed to memorization rather than learning. Thus, in all the experiments conducted using PhaGCN, we use genome-masking, meaning that the genomes in the testing data will be removed from the training data so that they do not share any genomes. The test contigs thus represent novel phages.

We compare our results with three representative and widely used pipelines: vConTACT 2.0, POGs and ClassiPhage. In addition, as CNN itself can conduct classification, we also compared with the CNN model we trained for PhaGCN.

### 3.2 Experiments using simulated contigs

In this experiment, we randomly chose 20 genomes from each family as the testing species. The remaining genomes were used as the training set. Thus the testing data contain 160 genomes from all 8 families under *Caudovirales*. Then, we sampled contigs from each test genome by generating random starting positions. To estimate the impact of contig length on PhaGCN, we generated contigs in three length ranges: [4 kbp, 8 kbp], [8 kbp, 12 kbp] and [12 kbp, 16 kbp]. Each contig is generated using a random start position and a random length within each range. For each of the length range, we generated 10 contigs. Thus we have 1600 contigs for testing. Finally, we repeated this experiment for three times and recorded the average performance of the three experiments. In total, for each length range, 4800 contigs from 480 genomes were tested. We also recorded the results of using complete genomes as the test data. To have a fair comparison, we applied the same method to construct the training set (or reference database) and the test set for vConTACT 2.0, POGs and ClassiPhage.

**Fig. 4.**
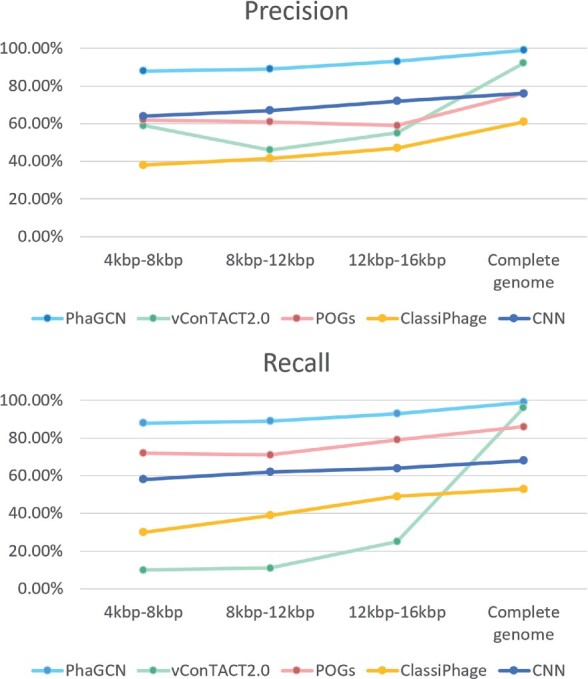
The precision and recall (Equations 13 and 14) of PhaGCN, vConTACT2.0, ClassiPhage, POGs and the CNN model in PhaGCN on simulated contigs, which are randomly sampled from phage genomes. *X*-axis: the length range of contigs. For each length range, there are 1600 randomly sampled contigs from 20 genomes of 8 families. The reported performance is averaged on three such sets of contigs for each length range

Figure 4 shows that PhaGCN outperforms other state-of-the-art tools across different length range. With the increase of contig length, the performance of all pipelines increases. This is expected because longer contigs contain more proteins, which can lead to better classification performance. We also evaluated the classification performance of only using the CNN model in PhaGCN. Both the recall and precision of using GCN is better than using only CNN, showing that the knowledge graph enhances the learning ability of the model. In addition, the classification performance of PhaGCN is stable with the change of the contig length, making it useful for classifying short contigs.

**Fig. 5.**
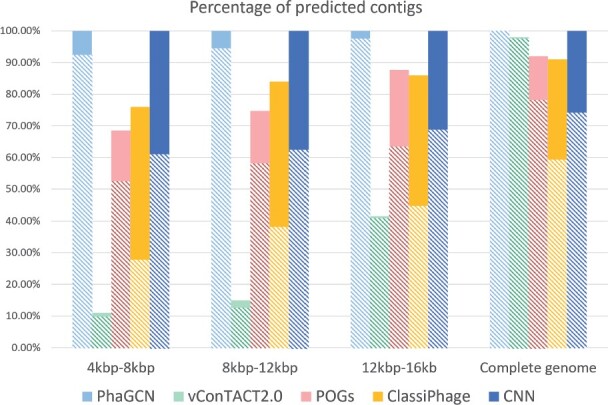
The percentage of classified contigs and classification accuracy (Equation 12) of each model on simulated contigs, which are randomly sampled from phage genomes. Each bar shows the percentage of classified contigs. The solid part shows the misclassification rate and the bottom part with patterns represents the macro-accuracy

Figure 5 shows the classification accuracy of all tools on randomly sampled contigs. The bar height shows the percentage of predicted contigs. In addition, each bar is divided into two parts. The top part (solid) is the misclassification rate while the bottom part with patterns corresponds to the macro-accuracy for the classified contigs. The result shows that PhaGCN always has the largest number of predicted contigs with the highest classification accuracy.

Although vConTACT 2.0 can assign the correct labels to most of the predicted contigs as shown in Figure 5, it only generated predictions for a small number of the contigs in two families (*Podoviridae* and *Siphoviridae*). For families without any prediction, the precision is 0. Thus, the macro-precision of vConTACT is small.

### 3.3 Experiments using simulated reads

In this experiment, we downloaded all newly released *Escherichia coli* phages under *Caudovirales* in 2020 from NCBI RefSeq and used them as the testing species (a total of 99 species are downloaded). And we downloaded all *Caudovirales* phages released in 2019 from NCBI RefSeq and used them as the training set. Consequently, these newly released *E.coli* phages can be treated as unknown phages for our model. Then we applied ART-Illumina to simulate reads from the testing sequences. The parameters used for generating reads are -p, -l 150, -ss HS25, -f 20, -m 200 and -s 10. The output contains 150 bp paired-end reads simulated under HiSeq 2500. We mixed all the simulated reads in one dataset and run MEGAHIT to assemble them. In order to quantify the performance of different tools, we determine the correct label of contigs by aligning them against the test genomes using BLAST in glocal mode. Only contigs of length above 2 kbp with taxon-specific alignment results and query coverage >85% will be kept. As a result, a total of 301 contigs were used as input for comparison.

**Fig. 6.**
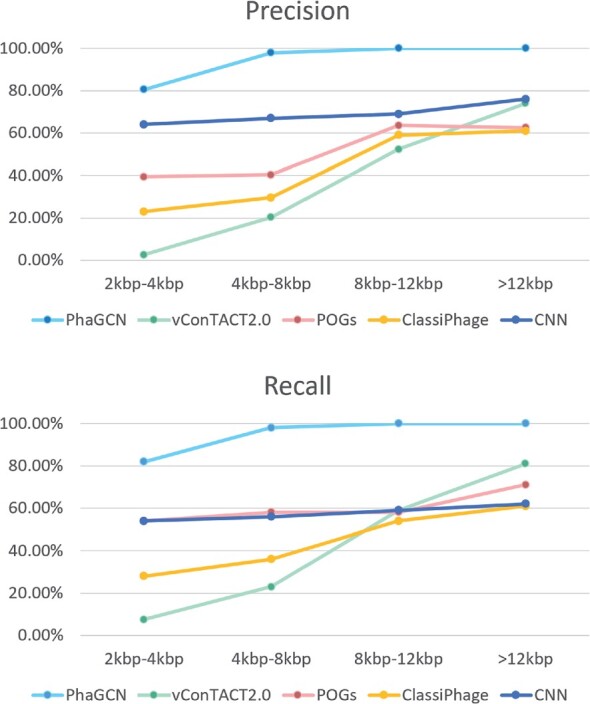
The precision and recall (Equations 13 and 14) of PhaGCN, vConTACT2.0, ClassiPhage, POGs and the CNN model in PhaGCN on contigs that are assembled using MEGAHIT from simulated reads. 301 contigs assembled from simulated reads of 99 species are used as inputs. The numbers of contigs for each length range are 101, 107, 51 and 42, respectively

**Fig. 7.**
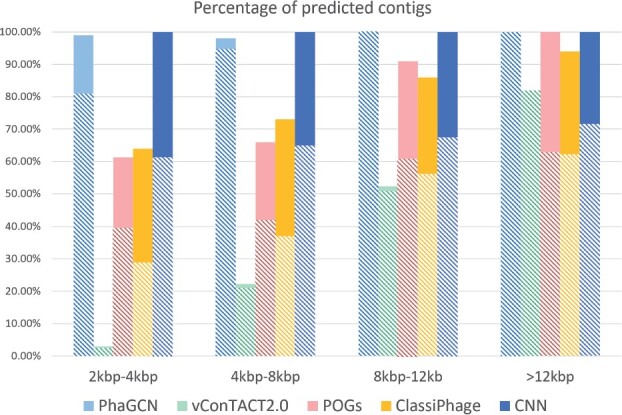
The percentage of classified contigs and classification accuracy (Equation 12) of each model on contigs that are assembled using MEGAHIT from simulated reads. Each bar shows the percentage of classified contigs. The solid part shows the misclassification rate and the bottom part with patterns represents the macro-accuracy

As shown in Figures 6 and 7, PhaGCN outperforms other tools across different length, which is consistent with the conclusion in Section 3.2. We also find that when the length of the contigs becomes shorter ([2 kbp, 4 kbp]), PhaGCN can still achieve over 80% accuracy. Although the reads simulated by ART-Illumina contain sequencing error, the performance of PhaGCN still achieves high accuracy (100% for contigs over 8 kbp). Because we only tested newly released *E.coli* phages in 2020, the classification performance of all methods were slightly better than the results in Section 3.2.

### 3.4 Running time comparison

The most resource demanding component in PhaGCN is the sequence alignment. We used it to produce the protein clusters by conducting pairwise alignments between contigs and reference sequences. PhaGCN produces protein clusters for each set of input contigs by assuming that they may contain novel proteins. In addition, alignment is also conducted for defining the edge in the knowledge graph. Table 2 shows the average elapsed time of classifying 100 contigs for each tool. PhaGCN is not the fastest program. Optimization can be applied to reduce the number of pairwise alignments. For example, we can produce a database of protein clusters and reduce the number of pairwise alignments.

**Table 2.** The average elapsed time to predict labels of 100 contigs for each method

Program	PhaGCN	vConTACT 2.0	ClassiPhage	POGs
Elapsed time (min/100 contigs)	12	32	4	7

*Note*: All the methods are run on Intel^®^ Xeon^®^ Gold 6258 R CPU with 8 cores.

### 3.5 Experiments on real sequencing data

In this experiment, we searched for real sequencing data that contain *Caudovirales* at NCBI SRA and downloaded two datasets, SRR12949983 and SRR13132427. Then we used MEGAHIT to assemble reads into contigs on these two datasets separately. To quantify the performance of phage classification on these two datasets, we used the provided read-level taxonomic analysis by NCBI SRA as the ground truth. The phages in the two datasets provided by NCBI SRA are listed in Table 3. We used the same method introduced in Section 3.3 to label the contigs and removed all these genomes in Table 3 from the reference database before training PhaGCN. The contigs in the test data are listed in Table 4.

**Table 3.** Reference species in SRR12949983 and SRR13132427

SRR12949983	SRR13132427
*Escherichia phage C5*	*Escherichia phage V18*
*Salmonella phage C2*	*Escherichia virus FV3*
*Serratia phage Pila*	*Escherichia virus JES2013*
*Escherichia virus E112*	*Escherichia phage CEC_Kaz_2018*
*Escherichia virus ECML134*	*Escherichia phage SECphi18*
*Escherichia virus T4*	*Escherichia phage vB_EcoS_PNS1*

*Note*: These species are shown in the associated taxonomic analysis at NCBI SRA. We use them as the ground truth to assign labels to the assembled contigs.

**Table 4.** Contigs assembled by MEGAHIT and their family labels derived from their alignments against the species in Table 3

SRR12949983		SRR13132427	
No. of contigs assembled by MEGAHIT (>2 kbp)			
24 (including 1 bacterial and 1 unknown)		20 (including 1 bacterial)	
Phage family	No. of contigs	Phage family	No. of contigs
*Autographiviridae*	5	*Myoviridae*	14
*Myoviridae*	17	*Siphoviridae*	5

**Fig. 8.**
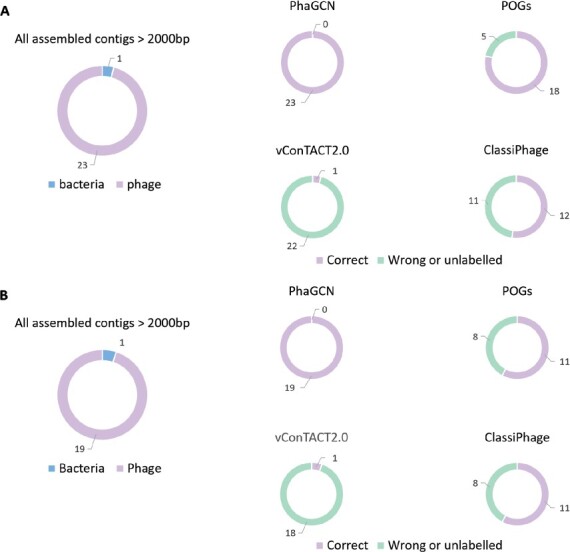
The classification result of PhaGCN, vConTACT 2.0, POGs and ClassiPhage on SRR12949983 (**A**) and SRR13132427 (**B**)

We compared the phage labels assigned by PhaGCN, vConTACT, POGs and ClassiPhage on the assembled contigs. Because the real sequencing data might contain bacteria sequences, we run DeepVirFinder (Ren et al., 2020) to reject the contigs that belong to bacteria. As shown in Figure 8, there is only one bacterial contig in each dataset. All other contigs (43 contigs) were fed into the four tools for classification.

Figure 8 reveals that PhaGCN achieves better classification performance than vConTACT, POGs and ClassiPhage. PhaGCN has 100% accuracy in both datasets. Many contigs could not be classified by vConTACT 2.0 (unlabeled in Figure 8). POGs and ClassiPhage are able to assign labels for all contigs but a number of them have wrong family labels.

In the dataset SRR121949983, there is one contig lacking ground truth because the contig cannot be aligned to any reference genome in Table 3. By extending the reference database to the Nucleotide Collection (nr/nt) database, BLAST shows that this contig belongs to a phage in *Siphoviridae*. We input this contig to the four tools and only PhaGCN assigned the correct label. It is worth noting that the reference genome is not in the training data of PhaGCN. The performance of alignment-based approaches heavily relies on the reference database while PhaGCN can learn the features for classifying new contigs.

**Fig. 9.**
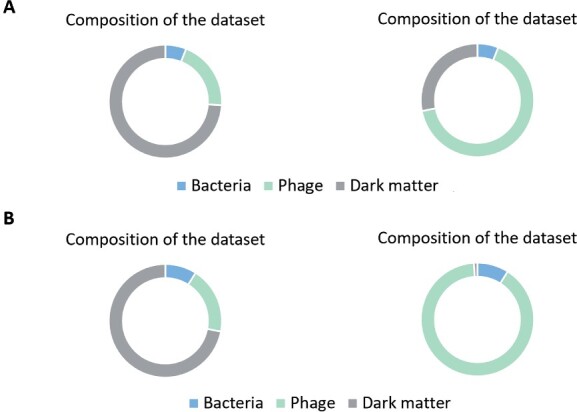
The composition analysis of SRR12949983 (**A**) and SRR13132427 (**B**). Left: composition analysis published at NCBI SRA. Right: composition analysis presented by PhaGCN

Furthermore, we showed the composition of these two datasets before and after using PhaGCN in Figure 9. The results revealed that our model can greatly improve the composition analysis for the dark matter. Thus, PhaGCN can benefit metagenomic analysis.

### 3.6 Phage classification in contigs produced from oyster metagenomic data

After validating PhaGCN on simulated datasets and two real sequencing datasets, we applied PhaGCN to contigs assembled from metagenomic data of oyster samples. The samples were collected by the co-author Dr. Jiang between April 2016 and July 2019 from various sites along the coast of South China Sea. Metagenomic sequencing was conducted from samples in the gill, visceral mass, and mantle tissues of oyster using viral-like particle enrichment and protocols in (Jingzhe and Hongying, 2018; Wei et al., 2018). There are about 2.5 billions of raw reads from 71 libraries.

After applying standard quality control and MEGAHIT with the default setting, there are about 3 375 091 contigs of length above 500 bp. After removing contigs that can be aligned to bacteria, archaea, eukaryota, we kept 22 966 contigs with length above 4000 bp as input to PhaGCN. Of them, 17 199 contigs can be assigned to *Caudovirales* by PhaGCN.

When users apply composition analysis for their samples, precision is important for generating valid hypothesis. Because vConTACT 2.0 has higher accuracy for what they can predict than ClassiPhage and POGs based on our experiments, we compared PhaGCN with vConTACT 2.0 in this experiment and summarized the results in Table 5. Although we don’t have the ground truth for this large-scale metagenomic sequencing data, the numbers of predicted contigs are consistent with the results of experiments on simulated and real sequencing datasets. The contigs vConTACT 2.0 can classify are significantly less than PhaGCN. ∼74.8% contigs are predicted by PhaGCN while ∼1.1% contigs are predicted by vConTACT 2.0. The contigs predicted by vConTACT is a subset of PhaGCN. We found that the clustering algorithm in vConTACT failed to group contigs and reference genomes in the same cluster. There exist many clusters containing only unlabeled contigs and thus, no labels will be assigned to these contigs. Also, PhaGCN can identify more families from the dataset. Because the classification accuracy of PhaGCN is more than 92% when the length of contigs is over 4 kbp, the result can provide useful family-level composition analysis for the oyster metagenomic data.

**Table 5.** Prediction results of PhaGCN and vConTACT for contigs produced from the oyster metagenomic data

	*Ackermannviridae*	*Autographiviridae*	*Demerecviridae*	*Drexlerviridae*	*Herelleviridae*	*Myoviridae*	*Podoviridae*	*Siphoviridae*	unclassified
vConTACT	0	0	0	0	0	50	116	102	22 698
PhaGCN	150	1727	301	74	32	5880	2682	6173	5767

### 3.7 Extension of PhaGCN

**Fig. 10.**
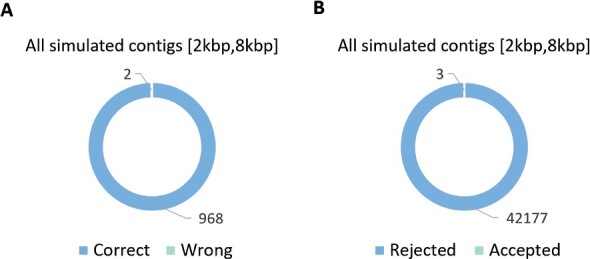
(**A**) The classification result of three added families: *Rudiviridae*, *Microviridae* and *Inoviridae*. (**B**) PhaGCN can reject non-*Caudovirales* phages

As *Caudovirales* is the order with the most number of sequenced phages from RefSeq, we validated PhaGCN on classifying families in this order. But PhaGCN can be conveniently extended to other taxa. We extended PhaGCN by adding families that contain at least 10 genomes. Using this criterion, three families (*Rudiviridae*, *Microviridae* and *Inoviridae*) were added. We used the leave-one-genome-out method to choose testing genomes from these three families and use the same method introduced in Section 3.2 to generate contigs. The classification results in Figure 10A show that PhaGCN can correctly classify almost all of the contigs from the extended families.

Using knowledge graph enables PhaGCN to detect targeted phage families, which is useful for applications where only some phages are of interest. Specifically, phages that are not in the training families usually won’t form edges with the nodes in the graph and thus will not be mis-classified into *Caudovirales*. We validated the detection ability of PhaGCN by testing whether PhaGCN can reject contigs that do not belong to *Caudovirales*. We downloaded 4218 phage genomes that do not belong to *Caudovirales* from RefSeq according to the ICTV taxonomic affiliation information. For each genome, we apply the same method introduced in Section 3.2 to generate 10 contigs for each of them. Thus, a total of 42 180 contigs were tested. As shown in Figure 10B, only 3 of them are accepted (predicted) by PhaGCN. This experiment demonstrates that PhaGCN can be applied for targeted phage detection.

## 4 Discussion

As shown in the experiments, the performance of alignment-based approaches, such as POGs and BLAST, heavily relies on the reference database. The ambiguous hits or lack of reference genomes for highly divergent or novel phages can decrease the classification accuracy. Existing learning-based tools like vConTACT 2.0 cannot achieve good performance on short contigs. In this work, we demonstrate that PhaGCN can render better performance for novel phage classification. The major improvement of our method stems from combined strength of the reference-based model and the learning-based model using the knowledge graph: the nodes contain automatically learned features from nucleotide sequences and the edges are created by protein-based alignment. Then the semi-supervised GCN is applied on the knowledge graph to utilize both labeled and unlabeled data for training.

Although PhaGCN has greatly improved phage contig classification, we have several goals to optimize or extend PhaGCN in our future work. First, we simplified the edge weight computation by assuming that all protein clusters can be chosen with the same probability. We will investigate whether incorporating protein cluster size and the cluster’s entropy in label distribution can render more accurate edges. Second, PhaGCN can reject non-relevant phages with high accuracy. Thus we will extend it to phage detection and compare it with DeepVirFinder. Third, although we have demonstrated that PhaGCN can be easily extended to more families, it is still hard to predict classes with only a few training samples (less than 10). We will incorporate relevant learning methods to improve the classification accuracy for small families. Finally, we will explore whether we can incorporate bacteria in our knowledge network for phage host detection. This can be used to further validate the classification results on the oyster metagenomic data.

## Funding

This work was supported by the Research Grants Council of the Hong Kong Special Administrative Region, China (Project No. CityU 11206819) and HKIDS (9360163) and NSF of China (31972847).

*Conflict of Interest*: none declared.

## References

[btab293-B1] Aiewsakun P. et al (2018) Evaluation of the genomic diversity of viruses infecting bacteria, archaea and eukaryotes using a common bioinformatic platform: steps towards a unified taxonomy. J. Gen. Virol., 99, 1331–1343. 30016225 10.1099/jgv.0.001110 PMC6230767

[btab293-B2] Alam T. et al (2020) Deep Learning in LncRNAome: contribution, challenges, and perspectives. Noncoding RNA, 6, 47. 33266128 10.3390/ncrna6040047 PMC7711891

[btab293-B3] Alipanahi B. et al (2015) Predicting the sequence specificities of DNA-and RNA-binding proteins by deep learning. Nat. Biotechnol., 33, 831–838. 26213851 10.1038/nbt.3300

[btab293-B4] Bazan J. et al (2012) Phage display—a powerful technique for immunotherapy: 1. Introduction and potential of therapeutic applications. Hum. Vaccin. Immunother., 8, 1817–1828. 22906939 10.4161/hv.21703 PMC3656071

[btab293-B5] Bolduc B. et al (2017) vConTACT: an iVirus tool to classify double-stranded DNA viruses that infect Archaea and Bacteria. PeerJ, 5, e3243. 28480138 10.7717/peerj.3243 PMC5419219

[btab293-B6] Buchfink B. et al (2015) Fast and sensitive protein alignment using diamond. Nat. Methods, 12, 59–60. 25402007 10.1038/nmeth.3176

[btab293-B7] Chibani C.M. et al (2019) Classifying the unclassified: a phage classification method. Viruses, 11, 195. 30813498 10.3390/v11020195 PMC6409715

[btab293-B8] Dutilh B.E. et al (2014) A highly abundant bacteriophage discovered in the unknown sequences of human faecal metagenomes. Nat. Commun., 5, 4498. 25058116 10.1038/ncomms5498 PMC4111155

[btab293-B9] Fernández L. et al (2018) Phage or foe: an insight into the impact of viral predation on microbial communities. ISME J., 12, 1171–1179. 29371652 10.1038/s41396-018-0049-5 PMC5932045

[btab293-B10] Huang W. et al (2012) ART: a next-generation sequencing read simulator. Bioinformatics, 28, 593–594. 22199392 10.1093/bioinformatics/btr708 PMC3278762

[btab293-B11] Hurwitz B.L. , U’Ren J.M. (2016) Viral metabolic reprogramming in marine ecosystems. Curr. Opin. Microbiol., 31, 161–168. 27088500 10.1016/j.mib.2016.04.002

[btab293-B12] Jang H.B. et al (2013) Phylogenomic network and comparative genomics reveal a diverged member of the ϕkz-related group, marine Vibrio phage ϕJM-2012. J. Virol., 87, 12866–12878. 24067958 10.1128/JVI.02656-13 PMC3838149

[btab293-B13] Jang H.B. et al (2019) Taxonomic assignment of uncultivated prokaryotic virus genomes is enabled by gene-sharing networks. Nat. Biotechnol., 37, 632–639. 31061483 10.1038/s41587-019-0100-8

[btab293-B14] Jingzhe J. , Hongying W. (2018). Isolation of viral like particles (VLP) from tissues of molluscs. *protocols.io*.doi: 10.17504/protocols.io.m4yc8xw.

[btab293-B15] Keegan K.P. et al (2016). MG-RAST, a metagenomics service for analysis of microbial community structure and function. In: Terry J. McGenity, Kenneth N. Timmis, Balbina Nogales Fernández (eds.) Microbial Environmental Genomics (MEG). Springer, New York, NY, pp. 207–233. 10.1007/978-1-4939-3369-3_13 26791506

[btab293-B16] Kipf T.N. , Welling M. (2016). Semi-supervised classification with graph convolutional networks. In: Kipf Thomas N. and Welling Max (eds.) International Conference on Learning Representations (ICLR). *arXiv preprint arXiv:1609.02907*.

[btab293-B17] Kristensen D.M. et al (2013) Orthologous gene clusters and taxon signature genes for viruses of prokaryotes. J. Bacteriol., 195, 941–950. 23222723 10.1128/JB.01801-12 PMC3571318

[btab293-B18] Li D. et al (2015) MEGAHIT: an ultra-fast single-node solution for large and complex metagenomics assembly via succinct de Bruijn graph. Bioinformatics, 31, 1674–1676. 25609793 10.1093/bioinformatics/btv033

[btab293-B19] Liu J. et al (2004) Antimicrobial drug discovery through bacteriophage genomics. Nat. Biotechnol., 22, 185–191. 14716317 10.1038/nbt932

[btab293-B20] Loc-Carrillo C. , Abedon S.T. (2011) Pros and cons of phage therapy. Bacteriophage, 1, 111–114. 22334867 10.4161/bact.1.2.14590 PMC3278648

[btab293-B21] McGrath S. et al (2007). Bacteriophage: Genetics and Molecular Biology. Caister Academic Press, Wymondham, UK.

[btab293-B22] Mikolov T. et al (2013). Distributed representations of words and phrases and their compositionality. In: Mikolov Tomas, Sutskever Ilya, Chen Kai, Corrado Greg, Dean, Jeffrey (eds.) Conference on Neural Information Processing Systems (NeurIPS). *arXiv preprint arXiv:1310.4546*.

[btab293-B23] Moon K. et al (2018) Genomic and ecological study of two distinctive freshwater bacteriophages infecting a Comamonadaceae bacterium. Sci. Rep., 8, 1–9. 29789681 10.1038/s41598-018-26363-y PMC5964084

[btab293-B24] Moon K. et al (2020a) Freshwater viral metagenome reveals novel and functional phage-borne antibiotic resistance genes. Microbiome, 8, 1–15. 32482165 10.1186/s40168-020-00863-4 PMC7265639

[btab293-B25] Moon K. et al (2020b) Viral metagenomes of Lake Soyang, the largest freshwater lake in South Korea. Sci. Data, 7, 1–6. 33051444 10.1038/s41597-020-00695-9 PMC7553992

[btab293-B26] Perez Sepulveda B. et al (2016) Marine phage genomics: the tip of the iceberg. FEMS Microbiol. Lett., 363, fnw158. 27338950 10.1093/femsle/fnw158 PMC4928673

[btab293-B27] Ren J. et al (2020) Identifying viruses from metagenomic data using deep learning. Quant. Biol., 8, 64–14. 34084563 10.1007/s40484-019-0187-4 PMC8172088

[btab293-B28] Rohwer F. , Edwards R. (2002) The Phage Proteomic Tree: a genome-based taxonomy for phage. J. Bacteriol., 184, 4529–4535. 12142423 10.1128/JB.184.16.4529-4535.2002 PMC135240

[btab293-B29] Santiago-Rodriguez T.M. , Hollister E.B. (2019) Human virome and disease: high-throughput sequencing for virus discovery, identification of phage-bacteria dysbiosis and development of therapeutic approaches with emphasis on the human gut. Viruses, 11, 656. 31323792 10.3390/v11070656 PMC6669467

[btab293-B30] Seo S. et al (2018) DeepFam: deep learning based alignment-free method for protein family modeling and prediction. Bioinformatics, 34, i254–i262. 29949966 10.1093/bioinformatics/bty275 PMC6022622

[btab293-B31] Shang J. , Sun Y. (2020). CHEER: hierarCHical taxonomic classification for viral mEtagEnomic data via deep leaRning. *Methods, 189, 95–103*. 10.1016/j.ymeth.2020.05.018 PMC7255349 32454212

[btab293-B32] Wang L.-F. , Yu M. (2004) Epitope identification and discovery using phage display libraries: applications in vaccine development and diagnostics. Curr. Drug Targets, 5, 1–15. 14738215 10.2174/1389450043490668

[btab293-B33] Wang Q. et al (2007) Naive Bayesian classifier for rapid assignment of rRNA sequences into the new bacterial taxonomy. Appl. Environ. Microbiol., 73, 5261–5267. 17586664 10.1128/AEM.00062-07 PMC1950982

[btab293-B34] Wei H.-Y. et al (2018) Detection of viruses in abalone tissue using metagenomics technology. Aquac. Res., 49, 2704–2713.

[btab293-B35] Zhao T. et al (2020) DeepLGP: a novel deep learning method for prioritizing lncRNA target genes. Bioinformatics, 36, 4466–4472. 32467970 10.1093/bioinformatics/btaa428

